# Bakterielle Impfstoffe für die Behandlung von rezidivierenden Harnwegsinfektionen: eine systematische Übersichtsarbeit und Metaanalyse

**DOI:** 10.1007/s00120-024-02444-x

**Published:** 2024-09-27

**Authors:** Fabian P. Stangl

**Affiliations:** Universitätsklinik für Urologie, Inselspital Bern, Freiburgstraße 41c, 3010 Bern, Schweiz

## Originalpublikation

Maka Q, Greig J, Dasgupta P, Malde S, Raison N (2024) Bacterial Vaccines for the Management of Recurrent Urinary Tract Infections: A Systematic Review and Meta-analysis. Eur Urol Focus. 10.1016/j.euf.2024.04.002

## Übersetzung

### Hintergrund.

Mehrere bakterielle Impfstoffe wurden entwickelt, um die sozioökonomische Belastung durch Harnwegsinfektionen (HWI) und den Einsatz prophylaktischer Antibiotika bei der Behandlung wiederkehrender HWI (rHWI) zu reduzieren. Diese systematische Übersichtsarbeit bewertet die Wirksamkeit von Impfungen bei der Prävention von rHWI.

### Methoden.

Medline, Embase und Web of Science wurden von Beginn der Aufzeichnung bis Dezember 2023 durchsucht. Kohortenstudien mit einer Vergleichsgruppe und randomisierte kontrollierte Studien (RCT), die die Wirksamkeit von Impfungen bei erwachsenen rHWI-Patienten gemäß vordefinierten Auswahlkriterien untersuchten, wurden inkludiert (PROSPERO-Registrierung: CRD42022356662). Eine gepoolte Analyse wurde für RCT durchgeführt, mit einer Untergruppenanalyse für Impfstofftypen und Booster-Regime. Andere Studien wurden narrativ dargelegt. Das Risiko eines Bias wurde mit dem Cochrane-risk-of-bias-Tool bewertet. Der GRADE-Ansatz („grading of recommendations, assessment, development, and evaluations“) wurde verwendet, um die Qualität der Evidenz zu bewerten.

### Ergebnisse und Einschränkungen.

Vierzehn Vergleichsstudien mit insgesamt 2822 Patienten, die fünf Impfstofftypen untersuchten, wurden ausgewählt. Das gepoolte Risikoverhältnis von 8 placebokontrollierten Studien für Patienten, die kurzzeitig (6–12 Monate) HWI-frei blieben, betrug 1,52 (95 %-Konfidenzintervall [KI] 1,05–2,20) mit einer erforderlichen Behandlungsanzahl von 6,45 (95 %-KI 2,80–64,80). Es besteht eine erhebliche Heterogenität und ein geringes Risiko einer Publikationsverzerrung.

### Schlussfolgerungen und klinische Implikationen.

Es gibt begrenzte Hinweise darauf, dass Impfungen kurzzeitig wirksam sind, um HWI bei erwachsenen weiblichen Patienten zu reduzieren. Aufgrund der niedrigen Qualität der Evidenz sind weitere langfristige RCT mit großen Stichprobengrößen erforderlich, um schlüssige Beweise für die langfristige Wirksamkeit von Impfungen bei der Prävention von rHWI zu liefern.

## Kommentar

Die vorliegende systematische Übersicht und Metaanalyse von Mak et al. untersucht die Wirksamkeit bakterieller Impfstoffe zur Prävention rezidivierender Harnwegsinfektionen (rHWI). HWI sind eine weit verbreitete Problematik, die insbesondere Frauen betrifft und mitunter zu rezidivierenden Episoden führt [1]. rHWI enden häufig in einer antibiotischen Therapie, was das Risiko für Antibiotikaresistenzen erhöht [2]. Die wachsende Sorge bezüglich multiresistenter Keime macht die Suche nach alternativen Präventionsstrategien dringend erforderlich. In diesem Kontext bieten bakterielle Impfstoffe eine vielversprechende Option, weil sie das Immunsystem gegen die häufigsten Erreger durch Exposition gegenüber bakteriellen Antigenen stärken. Die Analyse von Mak et al. liefert eine umfassende Bewertung der aktuellen Evidenzlage zur Wirksamkeit dieser Impfstoffe.

Die Autoren haben eine umfangreiche Literaturrecherche in den gängigen Datenbanken Medline, Embase und Web of Science durchgeführt und insgesamt 14 Vergleichsstudien mit 2822 Patienten in ihre Analyse identifiziert und einbezogen. Diese Studien umfassten sowohl randomisierte kontrollierte Studien (RCT) als auch Kohortenstudien mit Vergleichsgruppen. Die Evidenzqualität wurde mittels des GRADE-Ansatzes bewertet, um die Stärke und Zuverlässigkeit der Ergebnisse zu beurteilen.

Die gepoolte Analyse der 8 placebokontrollierten Studien zeigte, dass Patienten, die mit bakteriellen Impfstoffen behandelt wurden, eine signifikant höhere Wahrscheinlichkeit hatten, kurzzeitig (6–12 Monate) HWI-frei zu bleiben. Das relative Risiko (RR) betrug 1,52 (95 %-KI 1,05–2,20), was eine moderate Wirksamkeit nahelegt. Die notwendige Behandlungsanzahl („number needed to treat“, NNT) betrug 6,45 (95 %-KI 2,80–64,80). Diese Ergebnisse sind jedoch durch eine erhebliche Heterogenität (I^2^ = 77 %) zwischen den Studien eingeschränkt, was auf methodische Unterschiede und variierende Studienpopulationen hinweist.

Die GRADE-Bewertung ergab eine niedrige bis moderate Qualität der Evidenz. Hauptsächlich trugen methodische Schwächen, wie unklare Randomisierungsprozesse und variierende Definitionen von rHWI, zur Herabstufung der Evidenz bei. Darüber hinaus wurde ein geringes Risiko einer Publikationsverzerrung festgestellt, was die Interpretierbarkeit der Ergebnisse weiter einschränkt. Die Studien zur Bewertung der verschiedenen Impfstofftypen zeigten unterschiedliche Ergebnisse. Während für Uro-Vaxom und ExPEC4V keine statistisch signifikante Wirksamkeit nachgewiesen wurde, zeigten Studien zu Uromune vielversprechendere Ergebnisse. Diese Unterschiede könnten auf die Zusammensetzung der Impfstoffe zurückzuführen sein. Uro-Vaxom® und ExPEC4V bestehen aus verschiedenen Stämmen oder Antigenen von Escherichia coli, während Uromune mehrere hitzeinaktivierte Bakterienstämme enthält, darunter Escherichia coli, Klebsiella pneumoniae und Enterococcus faecalis. Es ist jedoch anzumerken, dass drei der vier verfügbaren Trials zu Uromune durch dieselbe Studiengruppe durchgeführt wurden. Hier wären größere multizentrische Untersuchungen notwendig und wünschenswert, um die Wirksamkeit auch in einer breiteren Population zu untermauern. Eine relevante Problematik aller Studien zu Impfstoffen bei HWI liegt in der eigentlichen Definition von HWI und der damit einhergehenden Heterogenität. Hier sollte für zukünftige Studien der Delphi-Consensus von Bilsen et al. [3] als Grundlage angewendet werden. Eine saubere und klar nachvollziehbare Einteilung von HWI reduziert hier eine mögliche Verzerrung und über- oder unterrapportieren von klinisch manifesten Infektionen. Dies würde auch eine stichhaltige Fallzahl- und Powerkalkulation erlauben. Die meisten eingeschlossenen Studien verfügen über eine begrenzte Anzahl von Patienten, was auf die hohe Anzahl von angenommenen Infekten im Patientenkollektiv zurückzuführen ist. Dieser Umstand ist eine häufige Problematik im Rahmen von Studien im Bereich der infektiologischen Forschung, weil historische Landmark-Trials z. B. im Bereich der Prostatastanzbiopsie eine sehr hohe Inzidenz von Infektionen publizieren. In der Folge kommt es zur oben genannten Verzerrung der Power-Kalkulation.

Ein weiterer wichtiger Aspekt ist die Sicherheitsbewertung der Impfstoffe. Die meisten Studien berichteten über eine geringe Inzidenz von Nebenwirkungen, die überwiegend mild waren. Schwere Nebenwirkungen waren selten, und keine der Studien berichtete über Todesfälle im Zusammenhang mit den Impfstoffen. Häufige Nebenwirkungen waren Kopfschmerzen, gastrointestinale Beschwerden und lokale Reaktionen an der Injektionsstelle.

Schlussendlich zeichnen sich auch bei dieser Metaanalyse die gängigen Mängel ab: Verschiedene Studientypen werden mit erheblicher Heterogenität zusammengefasst und analysiert, alte Trials mit hohem Verzerrungsrisiko müssen inkludiert werden, und insuffiziente Probandenzahlen führen zu minder belastbaren Ergebnissen. Diese Probleme sind nicht spezifisch für die vorliegende Studie, sondern stellen eine allgemeine Herausforderung für viele systematische Übersichtsarbeiten und Metaanalysen in diesem und anderen Bereichen der Medizin dar [4]. Klare Studienprotokolle sind notwendig, um solche methodischen Schwächen zu vermeiden und die Qualität der Evidenz zu verbessern. Standardisierte Definitionen und rigorose Studiendesigns würden die Vergleichbarkeit und Aussagekraft der Ergebnisse erheblich steigern und somit eine fundiertere Entscheidungsgrundlage für die klinische Praxis bieten.

### Fazit.

Die vorliegende Publikation liefert wertvolle Einblicke in die Wirksamkeit bakterieller Impfstoffe zur Prävention rezidivierender HWI. Die Studien zeigen, dass diese Impfstoffe eine vielversprechende Alternative zur Antibiotikaprophylaxe darstellen könnten, insbesondere angesichts der wachsenden Bedrohung durch Antibiotikaresistenzen. Obwohl die derzeitige Evidenz hauptsächlich auf kurzfristigen Ergebnissen beruht, sind die Sicherheitsprofile der Impfstoffe vielversprechend und rechtfertigen weitere Forschung.

Für Kliniker ist es wichtig, die potenziellen Vorteile bakterieller Impfstoffe zu berücksichtigen und auf zukünftige Forschungsergebnisse zu warten, um fundierte Entscheidungen über ihre Integration in die Praxis treffen zu können. Die Anwendung bakterieller Impfstoffe könnte einen wertvollen Beitrag zur Reduktion des Antibiotikaverbrauchs und zur Förderung eines verantwortungsvollen Umgangs mit Antibiotika leisten. Dies ist ein wichtiger Schritt hin zu einem besseren Antibiotic Stewardship und dem langfristigen Erhalt der Wirksamkeit von Antibiotika.

### Klinische Implikationen.

1. *Alternative zur Antibiotikaprophylaxe*: Bakterielle Impfstoffe bieten eine vielversprechende Alternative zur Antibiotikaprophylaxe.
2. *Kurzfristige Wirksamkeit*: Die Impfstoffe zeigen eine moderate Wirksamkeit in der kurzfristigen Prävention von rHWI (6–12 Monate). Langfristige Studien sind jedoch notwendig, um die Nachhaltigkeit dieser Wirkung zu bestätigen.
3. *Patientenselektion*: Diese Impfstoffe könnten besonders für Patientinnen von Vorteil sein, die häufig unter rHWI leiden und bei denen eine Antibiotikaprophylaxe aufgrund von Resistenzen oder Nebenwirkungen keine optimale Lösung darstellt.
4. *Sicherheitsprofil*: Die Sicherheitsprofile der untersuchten Impfstoffe sind vielversprechend, mit wenigen schwerwiegenden Nebenwirkungen.
5. *Forschung und Weiterentwicklung*: Weitere groß angelegte, methodisch hochwertige RCT sind erforderlich, um die langfristige Wirksamkeit und Sicherheit bakterieller Impfstoffe zu bestätigen.
